# Effects of Ethanol Concentration on Oral Aroma Release After Wine Consumption

**DOI:** 10.3390/molecules24183253

**Published:** 2019-09-06

**Authors:** Carolina Muñoz-González, María Pérez-Jiménez, Celia Criado, María Ángeles Pozo-Bayón

**Affiliations:** Instituto de Investigación en Ciencias de la Alimentación (CIAL), Consejo Superior de Investigaciones Científicas (CSIC)-Universidad Autónoma de Madrid (UAM), Campus de Excelencia Científica, 28049 Madrid, Spain

**Keywords:** wine, ethanol, intra-oral SPME, oral aroma release, aroma persistence

## Abstract

This paper evaluates, for the first time, the effects of ethanol concentration on the dynamics of oral (immediate and prolonged) aroma release after wine consumption. To do this, the intraoral aroma release of 10 panelists was monitored at two sampling points (0 and 4 min) after they rinsed their mouths with three rosé wines with different ethanol content (0.5% *v*/*v*, 5% *v*/*v* and 10% *v*/*v*) that were aromatized with six fruity esters (ethyl butanoate, isoamyl acetate, ethyl pentanoate, ethyl hexanoate, ethyl octanoate and ethyl decanoate). Overall, the results indicated that the extent of the effects of ethanol content on the oral aroma release were influenced by the subject, the ethanolconcentration and the type of aroma compound. This effect was also different in the immediate than in the prolonged aroma release. In the first in-mouth aroma monitoring, an increase in the ethanol content provoked a higher release of the more polar and volatile esters (ethyl butanoate, ethyl pentanoate), but a lower release for the more apolar and less volatile esters (ethyl octanoate, ethyl decanoate). Regarding the prolonged oral aroma release, an increase of ethanol content in wine increased the oral aroma release of the six esters, which might also increase the fruity aroma persistence in the wines. Future works with a higher number of individuals will be needed to understand the mechanisms behind this phenomenon.

## 1. Introduction

Ethanol, the most abundant volatile compound in wines and the second most abundant after water, is a determinant of its sensory quality [1]. The presence of ethanol has been related to different sensations, such as burning feeling or palate warming [2], physical and perceived viscosity [2] or sourness and sweetness balance [3], among others. Apart from its contribution per se, it has been suggested that ethanol concentration plays a significant role in the overall aroma perception in wines [4,5,6,7], although the mechanisms behind these effects are not completely known. However, the increasing interest of consumers for light, fruity, and low alcohol beverages has increased the necessity to deeper understand this phenomenon in order to promote new types of low alcoholic wines.

The studies about the effects of ethanol concentration on aroma compounds have been addressed through sensory and/or chemical approaches. From a sensory point of view, studies performed orthonasally (when a wine is smelled) have found that an increase in ethanol content provokes a decrease of the fruity and floral notes [4,5,6,7], while an increase in wood, pepper, or chemical notes [7], which would produce an odor imbalance. In this regard, in vitro static studies have shown that the presence of ethanol might change wine polarity which would modify the distribution of volatile compounds between the gas and liquid phases according to their physico-chemical properties (for a review on this topic see [1]). Although very relevant, these studies may not give a clear understanding of how the wine is actually perceived when it is consumed.

When a wine is consumed, a fraction of aroma compounds travel from the mouth to the olfactory receptors via retronasal after having undergone oral processing (swallowing, breathing, interaction with saliva, adsorption with mucus, etc.) [8,9,10,11]. This generates a pulse of aroma known as immediate aroma perception. Moreover, another aroma fraction remains in the oral cavity when the wine is no longer in the mouth, and being released over time, which is responsible for the prolonged aroma perception or aroma persistence. Therefore, the mechanisms involved in retronasal release and perception are dynamic and more complex than those behind orthonasal perception. In this regard, some retronasal sensory dynamic studies have found that ethanol increased the duration and intensity of floral notes [12]. In another study, the effect of ethanol on the olfactory intensity of specific wine volatile phenols has also been observed [13]. However, to our knowledge, there are no in vivo studies that have measured the effects of ethanol content on the partitioning and release of aroma compounds in the mouth considering the dynamics of wine consumption. This could be due, among others, to analytical problems related to the high abundance of ethanol compared to other volatiles of interest. To the authors’ knowledge, only one in vivo study has measured the effects of ethanol concentration on the retronasal aroma release in model wines [14] showing that the presence of ethanol increased the overall in vivo aroma release. This might be explained by changes in surface tension increases the surface area of wine coating the mouth during consumption, as well as by the Marangoni effect and the Rayleigh-Bénard convection described by Tsachaki and collaborators [15,16] using in vitro approaches. However, all these studies have been performed in model wines and have not taken into account the complexity of the wine matrix composition, which could have affected the observed effects. In this regard, Tsachaki and collaborators [16] showed that the in vitro aroma release in real wines was closer to that of an aqueous solution than to model wines. Therefore, to understand the effects of ethanol content on aroma release and perception, it is important to consider the real wine matrix composition and to use closer approaches to that of wine consumption.

With these antecedents in mind, the objective of this work was to study how ethanol concentration affects the dynamic (immediate and prolonged) oral aroma release in real wines. To this end, a dealcoholized rosé wine (0.5% *v*/*v* ethanol content) dosed with increasing amounts of ethanol (5% and 10%) was used. The three wines, aromatized with six fruity esters (4 mg/L), were evaluated by 10 panelists at two time points (30 sec “immediate aroma release” and 4 min after wine expectoration “prolonged oral release”) following the intra-oral SPME procedure for oral monitoring. These results will provide more information about how aroma release changes during consumption, and will be important to understand the role of ethanol on aroma persistence.

## 2. Results and Discussion

**Figure 1:**
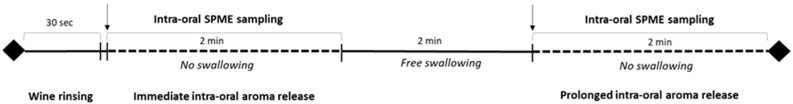
Sampling procedure followed for oral aroma monitoring.

To evaluate the effect of ethanol content on the in vivo aroma release during wine consumption, the oral aroma release of 10 panelists was monitored at two sampling points (Figure 1) after they rinsed their mouths with three rosé wines aromatized with six fruity esters (Table 1) presenting different ethanol content (0.5% *v*/*v*, 5% *v*/*v* and 10% *v*/*v*).

### 2.1. Effects of Ethanol Concentration and Individual Differences on the Immediate Oral Aroma Release

Immediate aroma release refers to the aroma compounds trapped in a SPME fiber immediately after the panelists rinsed their mouths with the wine samples and expectorated (Figure 1). Data obtained for the 10 volunteers in each of the wines were submitted to two-way ANOVA analyses considering individuals and ethanol content as factors and their interactions. Results (Table 2) showed that the effect of subjects on aroma release was highly significant (*p* < 0.0001) for all the assayed compounds (Table 2), which indicated a high interindividual variability on the immediate oral release of esters. The variability on the aroma released observed among individuals equally trained in the same consumption procedure and using the optimized SPME conditions is not surprising and it could be related to differences in oral physiological factors, such as, salivary flow rate and composition or oral cavity volume, among others, as it has been previously described in different works [8,9,10,11].

**Figure 2:**
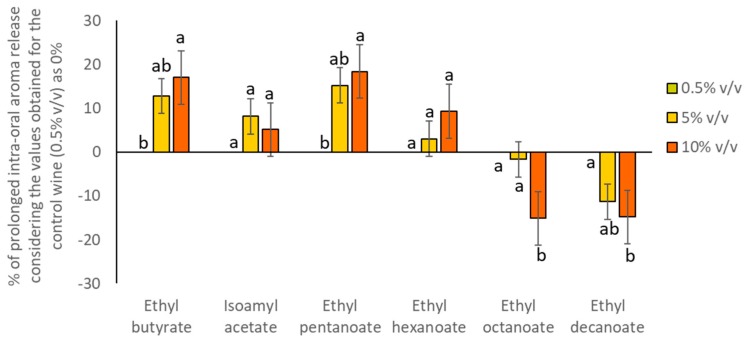
Percentage of immediate oral aroma release obtained for the volunteers (n = 10) after rinsing their mouths with the wines (considering the values obtained for the W 0.5% as 0% and comparing this value with the amount determined for the rest of wines). Different letters across the different compounds denote statistical differences after the application of the Tukey test for means comparison (*p* < 0.05).

Regarding the effect of ethanol content on the immediate oral aroma release, it seemed that this effect was less important than the subject effect and aroma compound and ethanol concentration-dependent (Table 2). To better visualize these results, data were plotted, and shown in Figure 2. To do so, data were normalized considering the values obtained for the 0.5% *v*/*v* as 0% and calculating the ratio for the other two wines. As can be seen, the oral release of two compounds (isoamyl acetate andethyl hexanoate) was not significantly affected by variations in the ethanol content. Moreover, certain compounds (ethyl butyrate and ethyl pentanoate) showed a significantly higher immediate oral release after the consumption of the wines with high ethanol content (5% *v*/*v* and 10% *v*/*v*) compared to 0.5% *v*/*v*, while others (ethyl octanoate and ethyl decanoate) showed the opposite behavior. The differences observed among compounds are not unexpected, and they could be related to the physicochemical properties of the aroma compounds, such as hydrophobicity (log P) or boiling point (BP), as it has been previously suggested by different authors [5,15,17,18,19]. Interestingly, the less polar and volatile compounds of this study (ethyl octanoate and ethyl decanoate) were less released in the wines with high ethanol content. This could be due to the fact that these compounds could be more soluble in the wine matrix as ethanol concentration increases. Therefore, the higher the ethanol content, the higher the retention of these compounds in the wine matrix and the lower the immediate oral release. On the contrary, the more polar and volatile compounds (ethyl butyrate and ethyl pentanoate) were more released in the wines with high ethanol content. These compounds would have had less affinity for the wine matrix as ethanol concentration increased, and therefore, they could have been more intra-orally released. However, the immediate release of the compound isoamyl acetate (showing a similar log P and BP values than those of ethyl pentanoate) was not significantly affected by the ethanol content. Interestingly, this is the only compound with a non-linear structure of the compounds assayed, which means that not only the phsysicochemical characteristics, but the structure of the compound could be important to explain oral aroma release.

**Figure 3:**
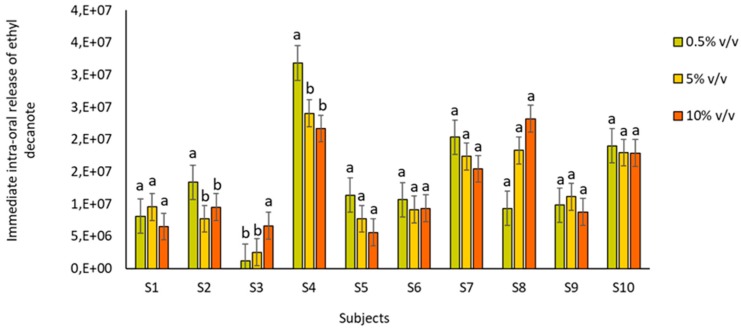
Immediate oral ethyl decanoate release (absolute peak areas) obtained for the volunteers (n = 10) after rinsing their mouths with the wines. Different letters across the different compounds denote statistical differences (*p* value < 0.05) after the application of the Tukey test for means comparison.

Finally, the interaction between subject and ethanol content was significant for all the aroma compounds assayed (Table 2). This means that the effect of ethanol on the immediate oral aroma release is individual dependent, and therefore, not all the panelists would be affected in the same way by changes in ethanol concentration. Figure 3 shows an example of the release values obtained for the compound ethyl decanoate by the 10 panelists. As can be seen, the variation in the ethanol content only significantly affected the oral aroma release of ethyl decanoate for three of the ten subjects (S2, S3 and S4). Moreover, the effects of ethanol content on oral aroma release showed opposite behaviors depending on the subject. While the presence of ethanol significantly reduced the release of ethyl decanoate for S2 and S4, it increased the release for S3. The different effects of ethanol concentration-depending on the subject are interesting, and it should be studied in future works considering a high number of individuals.

### 2.2. Effects of Ethanol and Individual Differences on the Prolonged Oral Aroma Release

To globally understand the effects of ethanol during wine consumption it is important to consider not only the aroma released immediately after wine intake, but also the oral aroma release that lingers in the mouth once the wine is no longer in the oral cavity. Therefore, after monitoring the immediate aroma release, the panelists were asked to keep their mouths closed and 2 min later, a second oral monitoring was carried out in the same conditions as described above (Figure 1). With these data, a two-way ANOVA analysis was performed in order to understand the effect of individual differences and ethanol content on the prolonged oral aroma release. Results are shown in Table 3. Similar to what happened in the immediate oral aroma release, a high variability among individuals and in the interactions between individuals and ethanol content was observed for all the aroma compounds assayed (Table 3).

**Figure 4:**
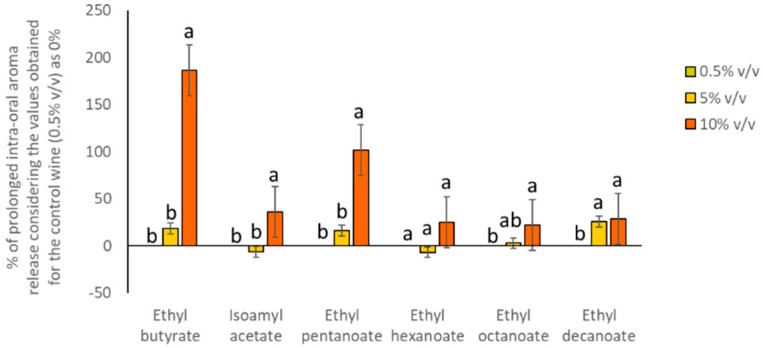
Percentage of prolonged oral aroma release obtained for the volunteers (n = 10) after rinsing their mouths with the wines (considering the values obtained for the W0.5% as 0% and comparing this value with the amount determined for the rest of wines). Different letters across the different compounds denote statistical differences after the application of the Tukey test for means comparison (*p* < 0.05).

Regarding the effects of ethanol content on the prolonged oral aroma release, it can be observed that the presence of ethanol significantly increased the oral release for five of the six esters assayed. Moreover, the release of ethyl hexanoate was also increased by the ethanol content, but the effect did not reach the significance level (*p*-value = 0.087) (Table 3). Once again, to better visualize these results, data were plotted, and shown in Figure 4. Here, data were normalized considering the values obtained for the 0.5% *v*/*v* as 0% and calculating the ratio for the other two wines. As can be observed, all the esters were more released at higher ethanol content. Moreover, the more apolar compounds (ethyl octanoate and ethyl decanoate) were also significantly more released in the 5% *v*/*v* than in 0.5% *v*/*v*. Interestingly, these results showed the same trend as the previous studies and dynamic sensory studies performed on this topic which showed an enhancement of aroma release in the presence of ethanol [14,20], and an increase in the duration of fruity/floral notes as the ethanol concentration increases [12].

Although additional studies are required in order to elucidate the mechanisms of action of ethanol content on the persistence of aroma compounds after wine intake, the higher prolonged oral aroma release observed in the presence of ethanol (from 5% *v*/*v* onwards) could be due to different facts. On the one hand, ethanol might induce changes in surface tension affecting the distribution of the wine in the mouth and pharynx during consumption, allowing the sample to better spread out and favoring the formation of a larger surface in the oral cavity for volatile release [20]. On the other hand, in the presence of ethanol, esters could be more solubilized in the layer of wine that remains in the mouth after wine rinsing and, therefore, these compounds could be released more slowly over time. Finally, the mixture of wine lingering in the oral cavity could be submitted to the Marangoni and Rayleigh convection phenomenon described in vitro by Tsachaki and co-authors [15]. In this case, the presence of ethanol would help aroma compounds be released more easily from the layer of wine to the oral headspace. Additionally, other mechanisms related to the interactions of ethanol with salivary proteins or enzymes could not be discarded. In this regard, it could be possible that the presence of ethanol could have inhibited certain salivary enzymes (e.g., esterases), more prone to metabolize long chain esters [21], like ethyl octanoate and ethyl decanoate. Additionally, the presence of ethanol (in higher concentrations than those of the aroma compounds) could have occupied the binding sites of the salivary proteins modifying the interactions between aroma compounds and oral proteins as suggested previously in the presence of sugar [22]. Therefore, additional studies are required in order to corroborate these hypotheses.

## 3. Material and Methods

### 3.1. Wine Samples

A dealcoholized rosé wine (Matarromera, Pesquera de Duero, Spain) from the Tempranillo grape variety with an ethanol concentration of 0.5% *v*/*v* (control wine) was selected for this study. To this wine, different amounts of ethanol content (Panreac Quimica S.A., Barcelona, Spain) were added to obtain two additional wines with 5% *v*/*v* and 10% *v*/*v* of ethanol content.

To reinforce the aroma profile of the wines, six food-grade esters (Sigma-Aldrich, Steinheim, Germany) were employed in this study. They present different physicochemical properties and are associated with fruity notes in wines (Table 1). Independent stock solutions of the compounds were prepared in food-grade ethanol (Panreac Quimica S.A., Barcelona, Spain). From here, each aroma compound was added to the wines immediately before each session to obtain a final concentration of 4 mg/L. It was previously determined by HS-SPME-GC/MS [23] that the amount of these compounds present in the original wine was negligible compared to the amount of aroma added (Table S1).

### 3.2. Intra-Oral SPME Sampling

#### 3.2.1. Subjects

Ten young, healthy volunteers (four male and six female) between 18–36 years old participated in this study. The sampling procedures were explained in detail to the subjects who also provided written consent to participate. The Bioethical Committee of the Spanish National Council of Research (CSIC) approved this study.

#### 3.2.2. Intra-Oral SPME Procedure for Aroma Monitoring

To study how ethanol affects the oral processing of typical wine esters, subjects rinsed their mouths with three aromatized wines containing different ethanol content (0.5% *v*/*v*, 5% *v*/*v* and 10% *v*/*v*), and then, the aroma released into the oral cavity after wine expectoration was monitored following the intra-oral SPME procedure previously developed and validated by Esteban-Fernandez and collaborators [24]. The representation of the analytical procedure is schematized in Figure 1. Briefly, subjects were instructed to introduce the aromatized wine (15 mL) into their oral cavity, kept it for 30 s and then spit it off. During rinsing, special care was taken to keep the lips closed, not to swallow and not to open the velum-tongue border prior to expectoration. At 30 s after expectoration, a DVB/CAR/PDMS (Divinylbenzene/Carboxen/Polydimethylsiloxane 50/30 μm thickness, 2 cm length) coated SPME fiber (Supelco, Bellefonte, PA, USA) with a home-made adaptor, was introduced into the oral cavity of the volunteer. After 2 min of oral aroma extraction, in which swallowing was not allowed, the fiber was removed from the oral cavity and immediately placed into the split/splitless injector (splitless mode) of the Gas Chromatograph (GC) Agilent 6890N coupled to a quadrupole Mass Detector (MS) Agilent 5973 (Santa Clara, CA, USA). This measurement is referred to as an immediate oral release. Four minutes after wine expectoration, a second fiber was introduced in the oral cavity following the exact protocol described previously (Figure 1). This second measurement is referred to as a prolonged oral aroma release.

After each injection, the fiber was cleaned for 10 min to avoid any memory effect. Analyses were performed three times with each wine type (0.5% *v*/*v*, 5% *v*/*v* and 10% *v*/*v*) by the ten volunteers (3 wine × 10 volunteers × 3 repetitions = 90 injections). An inter-fiber repeatability study was previously carried out. This allowed the selection of the most similar fibers (RSD values <10% for the extraction of the same aroma compound) to complete the study. Moreover, the use of a short sampling time also known as “true headspace” [25], avoids any problems derived from the use of SPME, such as the changes in the distribution of the molecules between the liquid and gas phases at equilibrium, due to adsorption phenomena onto the fiber itself, or to problems of saturation, due to an excess of ethanol absorbed onto the fiber compared to the compounds of interest [25]. In our case, the equilibrium is not reached, the fiber is not saturated, and therefore, the detected composition can be considered as representative of the real intraoral-headspace composition.

#### 3.2.3. GC/MS Analyses

Volatile compounds were separated on a DB-Wax polar capillary column (60 m × 0.25 mm i.d. × 0.50 um film thickness) from Agilent (J&W Scientific, Folsom, USA). Helium was the carrier gas at a flow rate of 1 mL min^−1^. The oven temperature was initially held at 40 °C for 2 min, then increased at 8 °C each minute to 240 °C and held for 15 min.

For the MS system (Agilent 5973N), the temperature of the transfer line, quadrupole and ion source were 270, 150 and 230 °C respectively. Electron impact mass spectra were recorded at 70 eV ionization voltages, and the ionization current was 10 µA. The acquisitions were performed in a scan (from 35 to 350 amu) and SIM modes. The identification of the six target compounds was based on the comparison of retentions times and mass spectra. The mass spectra were compared with those from NIST 2.0 database and with those from reference compounds analysed in the same conditions. Since no internal standard was used during the oral SPME extractions, absolute peak areas (APAs) were obtained. The use of APAs to express aroma release was sufficient for this type of analyses as the aim of the work was to compare the extent of aroma release between wine samples.

#### 3.2.4. Statistical Analyses

Two-way ANOVA and the Tukey test were used to determine significant differences in oral release parameters of the six aroma compounds considering subjects and wine type (ethanol concentration) as factors. The significance level was *p* < 0.05 throughout the study. The XL-Stat 2017.01 program was used for data processing (Addinsoft, Paris, France).

## 4. Conclusions

The present results showed that the effects of ethanol content on oral aroma release after wine consumption are dependent on the subjects, the ethanol concentration and their interactions and the nature of the aroma compound assayed.

The effects of ethanol content on oral aroma release were different immediately after wine consumption than four minutes later. In the first in-mouth aroma monitoring, an increase in the ethanol content provoked a higher release of the more polar and volatile esters (ethyl butanoate, ethyl pentanoate), but a lower release for the more apolar and less volatile esters (ethyl octanoate, ethyl decanoate). Regarding the prolonged oral aroma release, an increase of ethanol content in wine, increased the oral aroma release of the six esters. This effect was greater when the concentration of alcohol rose from 0.5% to 10% *v*/*v*, and it could be related to an increase of the fruity aroma persistence in the wines.

Moreover, important interindividual differences have been noted in this work, which could be related to variation in oral physiology parameters among subjects. Interestingly, high interaction between subject and ethanol was also found, meaning that in each subject, retronasal aroma release is differently affected by ethanol content. Future studies with a high number of individuals are needed in order to validate these results and to investigate the mechanisms behind the observed effects. Nonetheless, it is important to state that this research has shown the effect of ethanol on aroma release within the oral cavity but no information on the travel of aroma compounds to the receptors in the nose has been provided, which could be also of interest for new studies. Overall, these findings could help winemakers produce new low alcohol wines considering the effect of ethanol on aroma release.

## Figures and Tables

**Table 1:** Physicochemical properties of the aroma compounds employed in this study.

Aroma Compounds	CAS Number	MW ^a^ (g/mol)	log P ^b^	BP ^c^ (°C)	Solubility ^d^ (mg/L)	Aroma Descriptor ^e^
Ethyl butyrate	105-54-4	116.16	1.85	125.79	4900	Pineapple Strawberry
Isoamyl acetate	123-92-2	130.19	2.26	134.87	2000	Banana
Ethyl pentanoate	539-82-2	130.19	2.34	148.37	2210	Fruity
Ethyl hexanoate	123-66-0	144.22	2.83	170.05	309	Apple
Ethyl octanoate	106-32-1	172.27	3.81	210.70	70	Fruity apricot
Ethyl decanoate	110-38-3	200.32	4.79	247.43	16	Grape

^a^ Molecular weight. ^b^ Hydrophobic constant estimated using molecular modeling software EPI Suite (U.S. EPA 2000−2007). ^c^ Boiling point estimated using molecular modeling software EPI Suite (U.S. EPA 2000−2007). ^d^ Solubility in water estimated using molecular software EPI Suite (U.S. EPA 2000−2007). ^e^ From Flavornet database (http://www.flavornet.org; accessed October 2009) or PubChem (https://pubchem.ncbi.nlm.nih.gov/).

**Table 2:** Results of the two-way ANOVA performed to compare the statistical significance of the ethanol content on the immediate intra-oral aroma release (n = 10). Each sample was analyzed in triplicate.

		Ethyl Butyrate	Isoamyl Acetate	Ethyl Pentanoate	Ethyl Hexanoate	Ethyl Octanoate	Ethyl Decanoate
Mean		4,596,854.41	7,107,442.85	6,254,852.57	8,892,546.68	26,049,220.47	12,679,314.42
SEM ^a^		355,864.23	509,112.21	453,805.55	744,257.30	1,704,957.45	850,164.95
R² ^b^		0.94	0.94	0.91	0.87	0.95	0.93
F ^c^		25.27	24.43	17.228	11.47	30.10	21.29
Pr > F ^d^		<0.0001	<0.0001	<0.0001	<0.0001	<0.0001	<0.0001
Subject	F	56.14	59.49	40.55	31.37	78.78	58.32
	Pr > F	<0.0001	<0.0001	<0.0001	<0.0001	<0.0001	<0.0001
Ethanol (%)	F	4.04	1.06	3.73	0.57	6.75	4.29
	Pr > F	0.024	0.356	0.031	0.567	0.003	0.019
Subject*Ethanol (%)	F	12.30	9.57	7.17	3.31	8.54	4.65
	Pr > F	<0.0001	<0.0001	<0.0001	0.000	<0.0001	<0.0001

^a^ Standard error of mean. ^b^ Coefficient of determination. ^c^ F statistic. ^d^ Probability values.

**Table 3:** Results of the two-way ANOVA performed to compare the statistical significance of the ethanol content on the prolonged intra-oral aroma release (n = 10). Each sample was analyzed in triplicate.

		Ethyl Butyrate	Isoamyl Acetate	Ethyl Pentanoate	Ethyl Hexanoate	Ethyl Octanoate	Ethyl Decanoate
Mean		133,838.96	321,509.22	199,024.90	633,104.58	5,761,616.25	6,940,717.29
SEM ^a^		31,033.08	43,941.31	36,020.31	105,826.43	519,018.38	515,250.95
R² ^b^		0.83	0.96	0.86	0.90	0.91	0.91
F ^c^		9.02	45.07	11.32	21.13	19.41	18.74
Pr > F ^d^		<0.0001	<0.0001	<0.0001	<0.0001	<0.0001	<0.0001
Subject	F	19.80	121.52	31.67	63.78	55.75	48.91
	Pr > F	<0.0001	<0.0001	<0.0001	<0.0001	<0.0001	<0.0001
Ethanol (%)	F	8.91	12.64	7.44	2.56	3.76	7.55
	Pr > F	0.000	<0.0001	0.001	0.087	0.030	0.001
Subject*Ethanol (%)	F	3.62	10.38	1.66	1.98	2.82	4.54
	Pr > F	0.000	<0.0001	0.079	0.028	0.002	<0.0001

^a^ Standard error of mean. ^b^ Coefficient of determination. ^c^ F statistic. ^d^ Probability values.

## Supplementary Materials

The following are available online, Table S1: Endogenous concentration of the target aroma compounds in the control wine (0.5% *v*/*v*).
