# Supplementary material for: Effects of Ethanol Concentration on Oral Aroma Release After Wine Consumption
Source: Molecules. 2019 Sep 6;24(18):3253. doi: 10.3390/molecules24183253 (PMC6766967; doi:10.3390/molecules24183253)
Supplement: Supplementary file 1 [file molecules-24-03253-s001.pdf]

Supplementary Materials

Table S1. Endogenous concentration of the target aroma compounds in the control wine (0.5% v/v).

	Concentration (µg/L) in the control wine (0.5% v/v)	
Aroma compounds	**Mean**	**SD**
Ethyl butyrate	89.3	2.4
Isoamyl acetate	1.7	0.2
Ethyl pentanoate	n.d	-
Ethyl hexanoate	n.d	-
Ethyl octanoate	3.8	0.2
Ethyl decanoate	9.4	3.1
